# Pre-placement sharp angle traction-assisted endoscopic submucosal dissection for the treatment of laterally spreading tumors of the colon

**DOI:** 10.1055/a-2667-7377

**Published:** 2025-08-20

**Authors:** Jinpo Wang, Yunxin Chen, Miao Liu, Xiaoxiong Guo, Mingkai Zhuang, Fenglin Chen

**Affiliations:** 1 117890 Department of Gastroenterology, Fujian Medical University Union Hospital, Fuzhou, China; 2 Fujian Clinical Research Center for Digestive System Tumors and Upper Gastrointestinal Diseases, Fuzhou, China; 3 117890 Department of Gastrointestinal Endoscopy Nursing, Fujian Medical University Union Hospital, Fuzhou, China

Endoscopic submucosal dissection (ESD) is challenging in the colon due to limited submucosal space, as well as factors such as colonic folds and respiratory movements. Thus, traction-assisted endoscopy has gained attention 1 ; yet, the best traction technique remains under debate. The previously reported double-clip counter-traction method, while effective, faces limitations as the counter-traction effect diminishes and the submucosal space narrows during the procedure 2 . In this study, we refined the double-clip counter-traction technique and introduced a preplacement sharp angle traction method ( Video 1 ).

Preplacement sharp angle traction-assisted endoscopic submucosal dissection for a 2-cm sigmoid colon adenomatous polyp.Video 1

We encountered a 2-cm adenomatous polyp in the sigmoid colon, which exhibited lateral growth and a central depression. The procedure began with a submucosal injection, followed by a circumferential mucosal incision. Prior to applying traction, clip and a rubber band were strategically placed on the opposite side of the lesion to facilitate diagonally proximal traction ( Fig. 1 **a** ). An additional clip was utilized to secure the rubber band to the anal side of the lesion, creating a sharp angle of traction that elevated the submucosal tissue into a tent-like formation ( Fig. 1 **b** ). As dissection progressed, the traction effect gradually improved ( Fig. 1 **c** ). This method demonstrated successful application in the whole colon region.

**Fig. 1:**
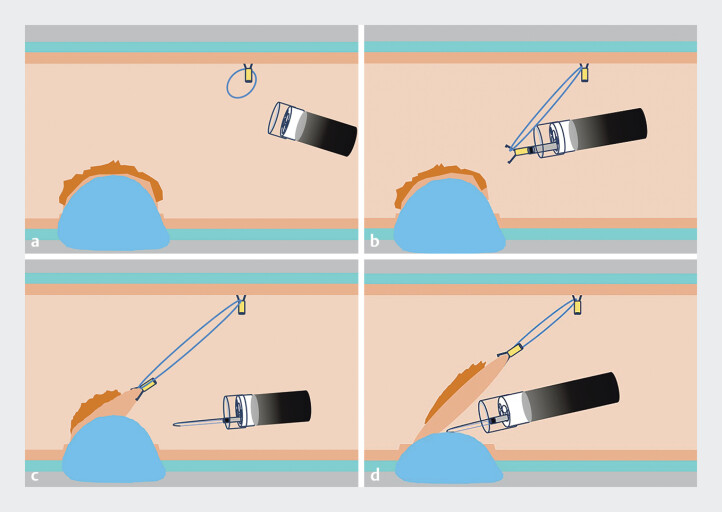
Schematic description of the strategy. **a** The clip with a rubber band anchored at an appropriate distance and orientation on the diagonally opposite of the lesion; **b** an additional clip was employed to secure the rubber band to the anal side of the lesion creating a sharp angle of traction; **c** good exposure of the submucosa tissue, which is easy to obtain satisfactory traction; and **d** integration with a transparent cap, and the traction effect progressively improves as the submucosal layer of the lesion is dissected.

Previous studies have indicated that positioning clips and rubber bands on the anal side of the lesion often fails to achieve an optimal traction angle. In contrast, the preplacement sharp angle traction method aligns better with the clinicianʼs expectations of the traction direction, allowing for easy adjustments in the event of positioning deviations. This tent-like traction technique not only enhances exposure of the submucosal tissue but also reduces the risk of muscular injury. Furthermore, this method integrates seamlessly with a transparent cap ( Fig. 1 **d** ), improving traction as the submucosal layer is dissected and facilitating rapid and complete resection of the lesion. Finally, pathological results confirmed R0 resection.

Endoscopy_UCTN_Code_TTT_1AQ_2AD_3AD

## Notes

Endoscopy E-Videos https://eref.thieme.de/e-videos
*E-Videos* is an open access online section of the journal *Endoscopy* , reporting on interesting cases and new techniques in gastroenterological endoscopy. All papers include a high-quality video and are published with a Creative Commons CC-BY license. Endoscopy E-Videos qualify for HINARI discounts and waivers and eligibility is automatically checked during the submission process. We grant 100% waivers to articles whose corresponding authors are based in Group A countries and 50% waivers to those who are based in Group B countries as classified by Research4Life (see: https://www.research4life.org/access/eligibility/ ). This section has its own submission website at https://mc.manuscriptcentral.com/e-videos .
